# Fruits and vegetables intake and its subgroups are related to depression: a cross-sectional study from a developing country

**DOI:** 10.1186/s12991-018-0216-0

**Published:** 2018-11-01

**Authors:** Elham Baharzadeh, Fereydoun Siassi, Mostafa Qorbani, Fariba Koohdani, Neda Pak, Gity Sotoudeh

**Affiliations:** 1 Department of Community Nutrition, School of Nutritional Sciences and Dietetics, Tehran University of Medical Sciences, Hojatdost Street, Naderi Street, KeshavarzBlv., Tehran, Iran; 2 Non-communicable Diseases Research Center, Alborz University of Medical Sciences, Karaj, Iran; 3 Department of Cellular, Molecular Nutrition, School of Nutritional Sciences and Dietetics, Tehran University of Medical Sciences, Tehran, Iran; 4 Shariati Hospital, School of Medicine, Tehran University of Medical Sciences, Tehran, Iran; 5 Children Hospital of Excellence, School of Medicine, Tehran University of Medical Sciences, Tehran, Iran

**Keywords:** Depression, Diet, Fruits, Vegetables, Women

## Abstract

**Background:**

The association of fruits and vegetables (FV) specific subgroups consumption and depression has not been investigated in healthy adult populations. Therefore, the aim of our study was to determine the relationship between intake of FV as well as their subgroups and depression.

**Methods:**

This cross-sectional study was conducted on 400 women attending healthcare centers. The scores of depression, anxiety, and stress were measured using the 21-item depression, anxiety and stress scales questionnaire. The participants’ anthropometric and physical activity data were collected and the 147-item semi-quantitative FFQ was used for estimating the FV intake.

**Results:**

After adjustment for confounding variables, the participants in the lower quartiles of total FV, total vegetables, total fruits, citrus, other fruits and green leafy vegetables intake were more likely to experience depression compared to those in the higher quartiles (*p* trend < 0.03).

**Conclusion:**

Our findings suggest that higher intake of total FV and some of its specific subgroups might be associated with depression.

## Background

According to the World Health Organization’s (WHO) report, depression is a common mental disorder. Globally, more than 300 million people of all ages suffer from depression. This disease is a major contributor to the overall burden of disease and is the leading cause of disability worldwide. Women are more affected by depression than men [1]. In Iran, the prevalence of mental disorders is 23.4% (27.5% in women and 19.2% in men), with the prevalence of depression being 10.3% (11.4% in women and 9.3% in men) [2]. Several studies have investigated the relationship between fruits and vegetables (FV) intake and development of depression, but the results are inconsistent. Cross-sectional studies have reported an inverse association between the consumption of FV and depression [3–7]. Some prospective cohort studies have also found that less FV consumption was associated with a higher risk of depression [8, 9]. A meta-analysis study revealed that consumption of FV is inversely related to the risk of depression [10]. Contrary to these results, some cross-sectional studies did not find any relationship between FV consumption and depression [11–13]. Further, in a prospective cohort study, it was found that only vegetables proved to be protective against symptoms of depression, while this effect was not found for fruits [14]. In some cross-sectional studies, an association was found between high consumption of vegetables [15], green leafy vegetables [16], and lower odds of depression, but no relationship was found for fruits [15, 16]. Nevertheless, for vegetable intake, no association was found in other studies [17, 18].

Oxidative stress [19, 20] and inflammation [21] are directly associated with the chance of depression. FV are rich in antioxidants and anti-inflammatory components, which may have beneficial effects in the prevention of depression [17]. Moreover, in FV, there are vitamins, especially folate, B6, C, E, and minerals, including calcium, iron, magnesium, potassium, dietary fiber and phytochemicals especially polyphenols. Vitamin E, C, and polyphenols, which have antioxidant properties, can reduce the oxidative stress. Most of the mentioned nutrients in FV may reduce inflammation, thereby reducing the risk of depression [22, 23]. In addition, FV-rich diets may increase the level of brain-derived neurotrophic factor (BDNF) which is an important protein for neural development and synaptic plasticity. Its low level will result in low mental health including depression [22].

Subgroups of FV have different contents of nutrients, fiber, antioxidants and phytochemicals [24]. Therefore, they may not affect the risk of depression equally. Therefore, it is better to evaluate individual FV or their specific subgroups.

Although the relationship between the intake of FV and depression has been reported in some studies, the specific subgroups of FV and their possible association with depression in apparently healthy population have remained understudied. A cross-sectional study found an inverse relationship between consumption of tomato, as well as tomato products and depression in elderly population [25]. Another cross-sectional study reported that higher consumption of raw FV predicted better mental health. However, this study was only limited to 18–25-year-old subjects [26]. To the best of our knowledge, apart from the mentioned study, no study has investigated the association of intake of FV subgroups and depression in healthy adult populations. Therefore, the aim of our study was to determine the relationship between the consumption of FV, as well as their specific subgroups and depression.

## Methods

### Subjects

This cross-sectional study was conducted on 400 women attending eight health centers in Khorramabad, Iran from May to October 2017. The inclusion criteria were ages 20–49 years, at least fifth grade elementary education, and body mass index of 18.5–34.9 kg/m^2^. On the other hand, the exclusion criteria were pregnancy and lactation, diagnosis of depression by a psychiatrist within a year prior to the start of the study, currently taking an antidepressant medication or in the past year, and use of tobacco or alcohol at least once a week. Women with diagnoses such as diabetes, cardiovascular disease, cancer, hypertension, kidney and liver disease, hyperthyroidism, epilepsy and MS, regular use of any medication or following special diet were excluded from the study. The objective and protocol of the study were explained to the participants, and informed consent was obtained from them before the study. The Ethics Committee of Tehran University of Medical Sciences approved the protocol of this study.

### Assessment of depression status

The Depression, Anxiety, Stress Scales (DASS, 21-items) questionnaire was used to measure the score of depression. This questionnaire was provided by Lovibond in 1995, which was validated by Afzali et al. for Iran [27, 28]. In the short form of the DASS questionnaire, for each subscale of depression, anxiety and stress, 7 questions have been presented. Individuals responded to each question based on to what extent that item applied to them during the last week (from 0 to 3: not at all, to some degree, to a considerable degree and very much, respectively). The scores on the DASS-21 were multiplied by 2 to calculate the final score. Based on the total score of depression, the subjects were divided into five groups of normal (0–9), mild (10–13), moderate (14–20), severe (21–27), and very severe (> 27) depression. However, due to the limited number of cases in some groups, they were simply divided into two groups of normal (< 10) and depressed (≥ 10) [29].

### Anthropometric and physical activity assessment

Weight was measured with minimal clothing and without shoes by a digital scale (Seca 813, Germany) with a measurement accuracy of 100 g. Their height was measured without shoes to the nearest 0.5 cm with a gauge. To calculate the body mass index (BMI), the weight was divided by the square of height (kg/m^2^). The International Physical Activity Questionnaire (IPAQ)—short form—was used to assess the physical activity of the participants [30]. Inquiries were made regarding vigorous and moderate activity and walking for at least 10 min/day during the previous 7 days. To calculate the activity, the duration and frequency of activity days were multiplied by the metabolic equivalent task value of the activity. The sum of the scores was calculated as the total physical activity per week.

### Dietary assessment

The usual dietary intake during the past year was assessed using a validated semi-quantitative food frequency questionnaire (FFQ) which included 147 food items [31]. In addition, some questions related to local spices and vegetables were added to the questionnaire. Finally, the number of questions reached 161 questions. The participants were asked to report their frequency of consumption of each food item during the previous year on a daily, weekly, or monthly basis. For estimating the daily food intake, the information of the FFQ was converted to g/day. The food items were analyzed for their energy and nutrients content using the Nutritionist IV software (First Databank, San Bruno, CA), modified for Iranian foods. The software database is drawn from United States Department of Agriculture (USDA) [32] food composition tables [33].

FV was divided into specific groups including cruciferous vegetables, green leafy vegetables, other vegetables, berries, citrus fruits, and other fruits based on previous studies [34, 35]. The Healthy Eating Index (HEI-2015) score [36] summarizes the consumption of 13 foods or nutrients including total fruits, whole fruits, total vegetables, greens and beans, whole grains, dairy products, total protein foods, seafood and plant proteins, fatty acids, refined grains, sodium, added sugars, and saturated fats. Each component was scored on a scale of 0–5 or 0–10. Similar to another study [24], we excluded FV when calculating the HEI score.

### Statistical analysis

Data analysis was performed using the SPSS version 16 (SPSS Inc.). The Kolmogorov–Smirnov test was used to evaluate the data normality. The data with an abnormal distribution were logarithmically transformed for statistical analyses. The geometric mean (standard error of mean) was calculated for the transformed data. The independent *t* test was employed to compare the quantitative variables between the two groups (depressed and healthy subjects). The mean values of the quantitative variables across the FV quartiles were then compared using the ANOVA test. Further, the ANCOVA test was utilized to compare the mean FV intake in the two groups after adjusting for potential confounders including BMI, energy intake, and HEI score. Variables with *p* value < 0.05 in ANOVA test were selected for adjustment. In addition, based on evidence from the literature, we selected age and physical activity as potential confounders [37]. Total vegetables and total fruits intake were mutually adjusted. For all of the vegetables and fruits sub-groups, FV intake was adjusted.

The relationship between FV intake and odds of depression was analyzed by simple logistic regression. In addition to the unadjusted analysis (Model 1), we used multivariable models to assess the relationship between FV intake and depression (Model 2). In Model 2, we adjusted for age, BMI, physical activity, energy intake, and HEI. Total vegetables and total fruits intake were mutually adjusted. For all of the vegetables and fruits subgroups, other subgroups of FV intake were also adjusted. Tests for trend were performed by introducing the categorical variables as continuous parameters in the models. *p* values less than 0.05 were considered significant.

## Results

The prevalence of mild, moderate, severe, and very severe depression was 10%, 8.5%, 5.8%, and 1%, respectively. The characteristics of the participants according to the quartiles of intake of total vegetables and fruits are presented in Tables 1, 2, and 3. With elevation of the total FV (Table 1), total vegetables (Table 2) and total fruits (Table 3) quartiles, the subjects had higher intake of energy, protein, carbohydrate, total fiber, percentage of energy from carbohydrate (*p* trend < 0.04), and lower intake of fat and percentage of energy from fat (*p* trend < 0.005). Upon increasing the total FV and total vegetables, the subjects had a higher percentage of energy from protein (*p*-trend < 0.002). In addition, with an elevation of the total FV and total fruits quartiles, the subjects had a higher HEI score (*p*-trend < 0.001).

**Table 1:** Characteristic of study participants according to quartiles of total vegetables and fruits intake

	Quartiles of total vegetables and fruits intake				
	1 (*n* = 100)	2 (*n* = 100)	3 (*n* = 100)	4 (*n* = 100)	*p*-trend^a^
g/day (median)	233.9	332.1	378.3	451.6	
Age (years)	33.3 ± 7	34.0 ± 7.7	34.2 ± 7.5	34.1 ± 7.1	0.4
Education (years)	12.1 ± 3.4	12.8 ± 3.1	12.5 ± 3.1	12.4 ± 3.8	0.6
BMI (kg/m^2^)	25.7 ± 3	26.2 ± 3.36	26.3 ± 2.6	25.5 ± 3	0.7
Physical activity (MET/h/week)	487.4 ± 275.6	473.1 ± 292.5	546.7 ± 247.9	500.7 ± 340.5	0.3
Height (cm)	161.6 ± 4.7	161.5 ± 4.7	161.8 ± 4.1	161.9 ± 4.7	0.5
Weight (kg)	66.8 ± 7.9	67.9 ± 9.6	68.8 ± 7.3	66.4 ± 7.7	0.9
Family size (n)	4.3 ± 1.3	4.5 ± 1.1	4.4 ± 1.3	4.4 ± 1.4	0.7
Dietary supplement use (%)	45 (45%)	49 (49%)	47 (47%)	53 (53%)	0.5^b^
Energy intake (kcal)	1579.6 ± 288.5^c^	1614.5 ± 266	1629.4 ± 289.6	1752.1 ± 355.6	< 0.001
Protein intake (g)	56 ± 9.5^c^	58.7 ± 10.1	59.5 ± 9.7	64.8 ± 11.5	< 0.001
Carbohydrate intake (g)	218.5 ± 47.1^c^	237 ± 44.5	240.1 ± 48.6	261 ± 57.4	< 0.001
Fat intake (g)	52.8 ± 13.7^c^	48 ± 11.3	48 ± 13.1	50.8 ± 15.2	0.003^d^
Energy of protein (%)	14.2 ± 1.2	14.6 ± 1.4	14.6 ± 1.3	14.8 ± 1.4	0.001
Energy of carbohydrate (%)	55.6 ± 5.3	58.9 ± 4.3	59.2 ± 5.2	59.7 ± 4.4	< 0.001^d^
Energy of fat (%)	30.6 ± 5.7	27.1 ± 4.6	27 ± 5.2	26.4 ± 4.5	< 0.001^d^
Total fiber intake (g)	38.9 ± 18.2^c^	42.8 ± 14.4	40.5 ± 16.8	46.8 ± 18.7	< 0.001^d^
Healthy eating index score	46.6 ± 5.3^e^	47 ± 4.8	48.3 ± 6.1	50.4 ± 7.6	< 0.001^d^

Values are means (standard deviations) or percentages unless stated otherwise

Vegetables include white cabbage, red cabbage, broccoli, cauliflower, spinach, lettuce, vegetable greens, carrot, yellow squash, cucumber, tomato, green squash, eggplant, celery, green peas, green beans, garlic, onion, bell peppers, turnip, mushroom, green peppers, olive, corn, leek and artichoke

Fruits include strawberry, blackberry, mulberry, orange, tangerine, sweet lemon, sour lemon, dried berries, grapefruit, cantaloupe, melon, pears, apricot, cherry, apple, plum, peach, persimmon, nectarine, greengage, figs, kiwi fruit, pomegranate, date, plum, sour cherry, banana, apple juice, cantaloupe juice, grapes, raisin, dry peach and apricot, fruit juice

^a^ANOVA

^b^Chi-square test

^c^Geometric mean ± SEM

^d^Kruskal–Wallis test

^e^Median ± interquartile range

**Table 2:** Characteristic of study participants according to quartiles of total vegetables intake

	Quartiles of total vegetables intake				
	1 (*n* = 100)	2 (*n* = 100)	3 (*n* = 100)	4 (*n* = 100)	*p*-trend^a^
g/day (median)	119.6	170.6	200.6	251.7	
Age (years)	34.3 ± 7.5	32.7 ± 7.1	34.3 ± 7.4	34.3 ± 7.1	0.5
Education (years)	12 ± 3.6	13 ± 3	12.7 ± 3.4	12.2 ± 3.4	0.8
BMI (kg/m^2^)	25.9 ± 3.2	25.6 ± 2.6	25.9 ± 3.1	26.4 ± 3.1	0.1
Physical activity (MET/h/week)	497 ± 292.4	510 ± 273.2	473 ± 256.8	527.8 ± 338.1	0.6
Height (cm)	161.4 ± 4.7	161.7 ± 4.9	162 ± 4.3	161.8 ± 4.4	0.4
Weight (kg)	66.7 ± 7.4	66.9 ± 8	68 ± 9	68.3 ± 8.5	0.1
Family size (n)	4.5 ± 1.2	4.3 ± 1.3	4.4 ± 1.3	4.4 ± 1.2	0.8
Dietary supplement use (%)	50 (50%)	46 (46%)	57 (57%)	41 (41%)	0.5^b^
Energy intake (kcal)	1600.3 ± 276.5^c^	1606.6 ± 289.7	1632.7 ± 274.5	1734.4 ± 366.0	0.001
Protein intake (g)	56.9 ± 9.1^c^	58.0 ± 10.5	59.6 ± 8.9	64.5 ± 12.2	< 0.001
Carbohydrate intake (g)	224.6 ± 50.7^c^	238.4 ± 47.1	240.6 ± 50.1	251.9 ± 56.1	< 0.001
Fat intake (g)	51.7 ± 13.3^c^	47.0 ± 11.5	48.4 ± 11.4	52.7 ± 16.5	0.004^d^
Energy of protein (%)	14.2 ± 1.4	14.5 ± 1.2	14.6 ± 1.2	14.9 ± 1.4	< 0.001
Energy of carbohydrate (%)	56.4 ± 6	59.5 ± 4.2	59.1 ± 4.7	58.3 ± 4.7	< 0.001^d^
Energy of fat (%)	29.7 ± 6.1	26.6 ± 4.3	27.1 ± 5	27.7 ± 5	< 0.001^d^
Total fiber intake (g)	40.8 ± 18.4^c^	42.3 ± 16.8	40.9 ± 17.0	44.7 ± 16.9	0.034^d^
Healthy eating index score	46.9 ± 5.6^e^	48.7 ± 6.6	47.6 ± 4.5	49 ± 7.5	0.06^d^

Values are means (standard deviations) or percentages unless stated otherwise

Total vegetables are defined as in Table 1

^a^ANOVA

^b^Chi-square test

^c^Geometric mean ± SEM

^d^Kruskal–wallis test

^e^Median ± interquartile range

**Table 3:** Characteristic of study participants according to quartiles of total fruits intake

	Quartiles of total fruits intake				
	1 (*n* = 100)	2 (*n* = 100)	3 (*n* = 100)	4 (*n* = 100)	*p*-trend^b^
g/day (median)	103.7	177.8	229.6	229.6	
Age (years)	34 ± 7.3	33.4 ± 7.7	34.8 ± 6.8	33.4 ± 7.3	0.8
Education (years)	12.1 ± 3.5	12.8 ± 3.1	12.2 ± 3.3	12.7 ± 3.6	0.3
BMI (kg/m^2^)	26.3 ± 3	25.8 ± 3.1	26 ± 2.7	25.6 ± 3.1	0.1
Physical activity (MET/h/week)	474.7 ± 287.9	495.5 ± 235.7	515.9 ± 282.7	521.8 ± 349.6	0.2
Height (cm)	161.8 ± 4.6	162.1 ± 4.4	161 ± 4.3	161.9 ± 4.9	0.6
Weight (kg)	68.5 ± 8.8	67.6 ± 9	67.1 ± 6.9	66.7 ± 8.1	0.1
Family size (n)	4.3 ± 1.3	4.3 ± 1.2	4.6 ± 1.1	1.4 ± 4.3	0.7
Dietary supplement use (%)	37 (37%)	56 (56%)	42 (42%)	59 (59%)	0.5^c^
Energy intake (kcal)	1603 ± 273.3^d^	1561.3 ± 254.6	1689.6 ± 350.8	1721.8 ± 319.2	0.001
Protein intake (g)	57.0 ± 9.9^d^	57.0 ± 9.6	62.2 ± 11.5	62.8 ± 10.3	0.001
Carbohydrate intake (g)	218.2 ± 45.3^d^	227.9 ± 42.9	250.7 ± 51.2	260.3 ± 55.6	0.001
Fat intake (g)	56.8 ± 12.6^d^	48.2 ± 10.5	50.9 ± 16.4	49.6 ± 12.6	< 0.001^e^
Energy of protein (%)	14.3 ± 1.4	14.6 ± 1.3	14.7 ± 1.1	14.6 ± 1.4	0.051
Energy of carbohydrate (%)	54.7 ± 5	58.5 ± 4.2	59.5 ± 4.5	60.6 ± 4.5	< 0.001^e^
Energy of fat (%)	31.6 ± 5.6	27.5 ± 4.5	26.4 ± 4.5	25.6 ± 4.4	< 0.001^e^
Total fiber intake (g)	42.7 ± 16.4^d^	43.5 ± 15.7	45.6 ± 16.3	50.6 ± 19.6	< 0.001^e^
Healthy eating index score	47.1 ± 5.3^f^	46.6 ± 4.7	47.4 ± 6.9	50.4 ± 6.0	< 0.001^e^

Values are means (standard deviations) or percentages unless stated otherwise

Total fruits were defined as in Table 1

^a^ANOVA

^b^Chi square test

^c^Geometric mean ± SEM

^d^Kruskal–Wallis test

^e^Median ± interquartile range

The comparison of FV intake between depressed and healthy subjects is presented in Table 4. The intake of total FV and all of their subgroups were significantly lower in depressed subjects as compared to their healthy counterparts (*p* < 0.02). After adjustment for confounding variables, these differences remained significant for total FV, total vegetables, cruciferous vegetables, other vegetables, total fruits, citrus fruits, and other fruits (*p* < 0.02).

**Table 4:** Fruits and vegetables intake across depressed and healthy subjects

Daily intake (g)	Depressed (*n* = 101) Mean ± SD	Healthy (*n* = 299) Mean ± SD	*p* ^a^	*p* ^b^	*p* ^c^
Total vegetables and fruits	289.1 ± 104.7	373.2 ± 76.4	< 0.001	< 0.001	< 0.001
Total vegetables	160.2 ± 69.1	197.1 ± 54.9	< 0.001^d^	< 0.001	< 0.001
Cruciferous vegetables	4.0 ± 5.0	6.5 ± 6.5	< 0.001^d^	< 0.001	0.005
Green leafy vegetables	16.3 ± 13.9^e^	23.9 ± 12.6	< 0.001	< 0.001	0.06
Dark yellow vegetables	5.1 ± 4.1	6.2 ± 4.5	0.01	0.011	0.1
Other vegetables	130.6 ± 55.2	157.9 ± 45.4	< 0.001^d^	< 0.001	0.016
Total fruits	118.5 ± 53.7^e^	170.6 ± 48.6	< 0.001	< 0.001	< 0.001
Berries fruits	0.5 ± 0.5	0.8 ± 0.6	< 0.001^d^	< 0.001	0.1
Citrus fruits	25.3 ± 12.5	35.2 ± 10.9	< 0.001^d^	< 0.001	< 0.001
Other fruits	93.8 ± 45.9^e^	134.4 ± 44.5	< 0.001	< 0.001	< 0.001

Total vegetables and fruits are defined as in Table 1

Cruciferous vegetables include white cabbage, red cabbage, broccoli, cauliflower

Green leafy vegetables include spinach, lettuce, and green vegetables such as basil, parsley, cress, leek, spearmint, origany, coriander and scallion

Dark yellow vegetables include carrot, yellow squash

Other vegetables include cucumber, tomato, zucchini, eggplant, celery, green pea, green bean, garlic, onion, green pepper, bell peppers, turnip, mushroom, olive, corn and artichoke

Berries fruits include strawberry, white mulberry black mulberry and dried berries

Citrus fruits include orange, tangerine, grapefruits, sweet lemon, sour lemon, orange juice

Other fruits include cantaloupe, melon, pear, apricot, cherry, apple, peach, nectarine, greengage, fig, kiwi fruit, persimmon, pomegranate, date, plum, sour cherry, banana, pineapple, grapes, dry peach and apricot, raisin and fruit juice

Values are means (standard deviations)

^a^Unadjusted, Student *t* test

^b^Adjusted for energy intake; ANCOVA test

^c^Adjusted for age, body mass index, physical activity, energy intake and healthy eating index score. Total vegetables and total fruits intake were mutually adjusted. For each vegetables and fruits sub-groups, total fruits and vegetables intake was adjusted; ANCOVA test

^d^Mann–Withney test

^e^Geometric mean ± SEM

The odds ratio (OR) of depression across quartiles of FV intake, before and after adjustment for confounding factors, is presented in two different models in Table 5. Before adjusting the confounders, the subjects in the lowest quartile of total FV intake and its all subgroups had a higher OR of depression compared with those in the highest quartile (*p* trend < 0.04). After adjusting for confounders, the participants in the lowest quartile of total FV, total vegetables, total fruits, citrus, other fruits and green leafy vegetables had a higher OR of depression compared with those in the highest quartile (*p*-trend < 0.03). Finally, the participants in the first quartile of berries (*p* = 0.01) had a higher OR compared with those in the highest quartile. However, no dose–response relationship was observed.

**Table 5:** Odds ratio (95% CI) of depression according to quartiles (Q) of fruits and vegetables intake

Daily intake	Q1	Q2	Q3	Q4	*p*-trend*
Total vegetables and fruits g/day (median)	233.9	332.1	378.3	451.6	
Cases of depressed	55	19	17	10	
Model 1 *p*	11 (5.12–23.59) < 0.001	2.1 (0.92–4.8) 0.07	1.84 (0.79–4.25) 0.1	1	< 0.001
Model 2 *p*	18.83 (7.96–44.51) < 0.001	2.98 (1.23–7.2) 0.01	2.47 (1.02–5.96) 0.04	1	< 0.001
Total vegetables g/day (median)	125.4	176.6	203.2	245.8	
Cases of depressed	54	16	14	17	
Model 1 *p*	5.73 (2.98–11.01) < 0.001	0.93 (0.44–1.96) 0.8	0.79 (0.36–1.71) 0.5	1	< 0.001
Model 2 *p*	4.43 (2.06–9.51) < 0.001	1.14 (0.50–2.59) 0.7	1.07 (0.46–2.5) 0.8	1	0.001
Cruciferous vegetables g/day (median)	2.5	3.1	6.2	6.2	
Cases of depressed	44	24	22	11	
Model 1 *p*	4.92 (2.33–10.42) < 0.001	1.79 (0.82–3.91) 0.1	1.53 (0.69–3.37) 0.2	1	0.001
Model 2 *p*	1.6 (0.57–4.48) 0.3	0.94 (0.34–2.55) 0.9	1.29 (0.52–3.21) 0.5	1	0.6
Green leafy vegetables g/day (median)	14.3	21.6	24.8	31.6	
Cases of depressed	49	17	15	20	
Model 1 *p*	3.84 (2.05–7.19) < 0.001	0.81 (0.4–1.67) 0.5	0.7 (0.33–1.47) 0.3	1	< 0.001
Model 2 *p*	1.61 (0.64–4.02) 0.3	0.55 (0.22–1.35) 0.1	0.58 (0.24–1.4) 0.2	1	0.02
Dark yellow vegetables g/day (median)	2.1	4.3	5.7	7.7	
Cases of depressed	34	25	20	22	
Model 1 *p*	1.82 (0.97–3.42) 0.06	1.18 (0.61–2.27) 0.6	0.88 (0.44–1.57) 0.7	1	0.03
Model 2 *P*	0.45 (0.18–1.09) 0.07	0.61 (0.26–1.41) 0.2	0.68 (0.3–1.53) 0.3	1	0.2
Other vegetables g/day (median)	102.7	144.6	166.2	190.3	
Cases of depressed	49	24	9	19	
Model 1 *p*	4.09 (2.17–7.73) < 0.001	1.34 (0.68–2.65) 0.3	0.42 (0.18–0.98) 0.04	1	< 0.001
Model 2 *p*	2.05 (0.88–4.79) 0.09	1.43 (0.64–3.23) 0.3	0.5 (0.19–1.33) 0.1	1	0.07
Total fruits g/day (median)	108.3	155.6	180	209.2	
Cases of depressed	58	17	14	12	
Model 1 *p*	10.12 (4.91–20.85) < 0.001	1.5 (0.67–3.33) 0.3	1.19 (0.52–2.72) 0.6	1	< 0.001
Model 2 *p*	11.08 (4.96–24.75) < 0.001	2.12 (0.90–5.01) 0.08	1.27 (0.54–2.99) 0.5	1	< 0.001
Berries fruits g/day (median)	0.4	0.5	0.5	0.6	
Cases of depressed	53	20	12	16	
Model 1 *p*	5.72 (2.95–11.11) < 0.001	1.33 (0.64–2.75) 0.4	0.69 (0.30–1.54) 0.3	1	0.001
Model 2 *p*	2.77 (1.24–6.18) 0.01	0.81 (0.34–1.91) 0.6	0.68 (0.27–1.68) 0.4	1	0.3
Citrus fruits g/day (median)	24.7	34.3	36.4	37.6	
Cases of depressed	55	22	12	12	
Model 1 *p*	8.96 (4.36–18.42) < 0.001	2.06 (0.96–4.45) 0.06	1 (0.42–2.34) 1	1	< 0.001
Model 2 *p*	3.14 (1.34–7.38) 0.008	1.27 (0.53–3.04) 0.5	0.68 (0.27–1.74) 0.4	1	0.004
Other fruits g/day (median)	85.1	121.1	141	167.4	
Cases of depressed	55	19	14	13	
Model 1 *p*	8.17 (4.04–16.52) < 0.001	1.57 (0.72–3.38) 0.2	1.08 (0.48–2.45) 0.8	1	< 0.001
Model 2 *p*	4.93 (1.97–12.31) 0.001	1.61 (0.67–3.89) 0.2	1.03 (0.43–2.51) 0.9	1	< 0.001

Model 1: unadjusted

Model 2: adjusted for age, body mass index, physical activity, energy intake and healthy eating index. Total vegetables and total fruits intake were mutually adjusted. For each vegetables and fruits sub-groups, other sub-groups of fruits and vegetables intake was adjusted

Fruits and vegetables are defined as in Tables 1 and 4

* Tests for trend were performed by entering the categorical variables as continuous parameters in the models

## Discussion

This study examined the relationship between the intake of various FV and depression among apparently healthy women. The results of this study suggested that a high intake of total FV, total vegetables, total fruits, citrus, other fruits and green leafy vegetables was independently related to a lower OR of depression. In addition, berries were inversely associated with depression, although no clear dose–response relationship was found.

Studies conducted to investigate the relationship between specific subgroups of FV and depression are very limited and there is no study in healthy adult populations. Our study provided evidence supporting benefits of consuming citrus, other fruits, green leafy vegetables and to a lesser extent berries.

Our findings are consistent with the results of other studies, suggesting an inverse relationship between FV consumption and depression. Cross-sectional studies [3–5, 7] and some prospective cohort studies [8, 9] have suggested that lower FV consumption was associated with a higher risk of depression. In addition, the inverse relationship between depression and fruits [9, 17, 18, 38] or vegetables [9, 15, 39, 40] consumptions has been separately indicated. A meta-analysis study indicated that intakes of both fruits and vegetables were inversely related to the risk of depression [10]. In subgroup analysis by the study design, higher fruits intake was associated to lower risk of depression in both cross-sectional and cohort studies. However, the association between higher vegetable intake and lower risk of depression was found in only cohort studies [11]. Contrary to these studies, the association between depression and FV intake or fruits and vegetables individually was not shown in other studies [3, 12–15, 17, 18, 32, 41, 42].

In some cross-sectional studies in elderly subjects, there was an association between high consumption of green leafy vegetables [16] as well as tomato and tomato products [25] and lower odds of depression. A case–control study which compared consumptions of food groups between depressed and healthy women revealed that subjects with more intake of citrus, berries, melons, other fruits, green leafy vegetables, yellow vegetables, and other vegetables had a lower chance of developing depression [43]. Since these are findings from a case–control study, the possibility that depression may influence the FV intake of subjects cannot be ruled out. In addition, other dietary components have not been controlled either.

Relevant data from clinical trials are scarce. Dietary improvements including increasing fruits and vegetables intake in patients with depression reduced the symptoms of depression [44]. However, in some other studies, higher intake of FV [45], concord grape juice [46] and blueberry juice [45] supplementation did not improve depression.

The biological mechanisms for the inverse association of FV intake and depression are not clear.

However, this association may be due to the large amount of bioactive compounds present in FV [24]. FV have a high content of micronutrients and phytochemicals including antioxidants and anti-inflammatory components which have detrimental effects on depression [17]. Antioxidants such as carotenoids, vitamin C, and vitamin E might prevent depressive symptoms [42]. High intake of B vitamins such as folic acid have been associated with lower risk of depression [47]. FV also supplies dietary fiber whose role in improving mood has been suggested [48]. Green leafy vegetables are good sources of folate and magnesium which are important in the prevention of depression. Folate is involved in the metabolism of monoamines such as serotonin in the brain [42]. Reduced synthesis of serotonin results to depressed mood [49]. In magnesium deficiency, high levels of calcium and glutamate reduce synaptic function and lead to depression [50]. In addition, lower levels of C-reactive protein, a marker of low-grade inflammation, has been reported in magnesium sufficiency [22]. In a study on rats, it was found that tomato juice inhibited monoamine oxidase (MAO) enzyme. This result suggested the anti-depressant properties of tomato [51]. In addition, Aronia melanocarpa berry juice with the highest polyphenol contents among fruits could yield an antidepressant effect in rats [52]. A study found that heptamethoxy flavone (HMF), a citrus flavonoid, increases the expression of BDNF in the hippocampus in rats, thereby exerting anti-depressant effects [53].

Women who were included in the present study were not informed of their depression status, which is one of the strengths of this study. When subjects are aware of their depression disorder, they might change their food intake. Another strength of our study was the adjustment for many confounding variables, especially the overall quality of diet. However, this study was limited in some aspects. First, there is a probability of error in answering FFQ questions. The reliability and validity of the utilized FFQ were not assessed for this population. Second, although we adjusted for many confounding variables, there may still be residual confounding variables which would affect our results. In addition, as with all cross-sectional studies, the present study reveals the existence or the absence of a relationship, while it does not specify causality. Further, FV-rich diets have been accompanied by a healthy lifestyle which may have not been adjusted in our analysis. Finally, our findings may not be generalized to other populations.

## Conclusions

In conclusion, we found that higher consumption of total FV, total vegetables, total fruits, citrus, other fruits, green leafy vegetables and berries might be associated with a lower OR of depression. The findings from this study support encouragement of FV consumption as part of a healthy diet and highlight the importance of FV consumption and a number of their subgroups in mitigating the chance of depression. Further studies focusing specifically on FV subgroups are required to confirm these findings.

## Abbreviations

FV: fruits and vegetables
BDNF: brain-derived neurotrophic factor
BMI: body mass index
DASS: Depression Anxiety Stress Scale
FFQ: food frequency questionnaire
WHO: World Health Organization
IPAQ: international physical activity questionnaire
HEI: healthy eating index
OR: odds ratio
MAO: monoamine oxidase
HMF: heptamethoxy flavone
